# The Digital Divide of Know-How and Use of Digital Technologies in Higher Education: The Case of a College in Latin America in the COVID-19 Era

**DOI:** 10.3390/ijerph19063358

**Published:** 2022-03-12

**Authors:** Judit García-Martín, Jesús-Nicasio García-Sánchez

**Affiliations:** 1 Department of Developmental and Educational Psychology, Universidad de Salamanca, 37008 Salamanca, Spain; 2 Department of Psychology, Sociology and Philosophy, Universidad de León, 24071 Leon, Spain; jn.garcia@unileon.es

**Keywords:** COVID-19, digital technologies, digital divide, higher education, Latin America

## Abstract

To determine whether or not digital inequalities exist, the frequency, duration, satisfaction, importance, and perceived competence of eighteen groups of digital tools during the COVID-19 pandemic confinement were examined. An online survey was administered to 2882 Latin American university students (49% female; M = 21.3 years). The following items are checked: (1) increased digital inequalities during the pandemic; (2) adequate reliability and validity of the Digital Technology Survey (DTS) instrument; (3) patterns of digital inequalities to the detriment of men, lower strata and unemployed people; and (4) evidence that the importance of digital activities as a causal factor on satisfaction with such digital activities as an outcome is mediated by the purpose of use and communication recipients, but not by strata or employment status, nor moderated by gender. The results are discussed in the light of previous studies, the limitations of the study and future perspectives.

## 1. Introduction

In March 2020, the COVID-19 coronavirus outbreak becomes a global pandemic [1,2,3,4], spreading rapidly to Europe, the United States and Latin America and causing disruptions in different sectors of human activity [2,4,5], and higher education is no exception [4,6,7]. In order to reduce this spread and mitigate, to the largest extent possible, its effects, the World Health Organization (WHO) has developed a public health guidance document and a strategic plan. According to this plan, forced confinement and social isolation measures have been applied in almost all countries of the world [2], leading to the temporary closure of Higher Education Institutions (HEIs) and the inevitable suspension of face-to-face teaching activities, which have been replaced by online teaching, affecting 94% of the world’s university population, as it modifies teaching and learning processes, causing a greater impact on those who lack the means and infrastructures at home [7].

Estimates by UNESCO IESALC (2020) show that some 23.4 million higher education students in Latin America and the Caribbean had been affected before the end of that month. This implies an urgent redesign of the usual teaching situations and switching to a virtual scenario, which represents an unprecedented challenge for university authorities and educational agents (teachers and students). Until then, online teaching was presented as exceptional and complementary to face-to-face teaching and was intended, in most cases, for one-off practices related to the implementation of innovative teaching methodologies [8], which implies three major challenges: infrastructure, administration and teaching.

This situation is characterised by apprehension and imprecision in the face of health developments and their economic and social consequences have increased digital inequalities [8,9,10], making the digital divide one of the problems worldwide, given the differences in access to technological resources and internet connection, especially in Latin American countries [11] and the lack of command in the use of digital tools [12,13]. In general, the online learning modality during the pandemic has shown the gap at three levels [14]: (i) access to electronic devices as well as internet connection, (ii) use of digital technologies at home and the quality thereof, and (iii) digital competences of teachers and students to make effective use of digital tools for learning purposes.

## 2. Literature Review: The Digital Divide

### 2.1. Background on Understanding the Third Level of the Digital Divide: Relearn to Learn

With the current epidemiological situation caused by the COVID-19 pandemic, as evidenced by [15], digital tools constitute the backbone and strategic axis of training and learning management. In this regard, [16] argues that the use of these digital technologies provides students with the means, resources, and content, but what is really important is that they give rise to learning situations where interactions and experiences of interconnection and educational innovation are promoted, ultimately favouring the involvement of students in the learning process as active agents. Furthermore, according to [17], one of the most relevant particularities of today’s society is undoubtedly the continuous use of digital technologies [12,18,19,20], turning them into elements of discrimination, and in the most extreme social contexts, of exclusion. In this sense, in its report, UNESCO includes the digital divide as one of the five obstacles to the rise of digital tools and the development of today’s knowledge and information society. For this reason, from that moment on, the digital divide became a priority objective. However, what is meant by the *digital divide* metaphor?

In line with the above, according to [21], the first-level divide (1995–2004) is understood as the inequality of access to information, knowledge and therefore to education and training through digital tools, whether for social, economic, age, gender, race, geographic location or other reasons. This is evidenced by the results obtained by [22], where it is stated that digital tools are a means of social exclusion in Mexico, as 50% of households do not have a computer, and more than 60% do not have access to the internet. This fact has experienced an increase because of the current pandemic. Similarly, in another study conducted in Spain by [23] during the COVID-19 pandemic among 593 students, it was found that 14% of them stated that they did not have the necessary technological resources to cope with the situation and to be able to continue carrying out their duties and activities. This is one of the reasons why 40.6% of the participants reported having to move to another place to spend the confinement.

The second-level divide (2004–2019), which is more sophisticated and multidimensional, focuses on the lack of skills and competences to use digital tools (digital literacy) rather than on access thereto.

Recently, however, a new level has emerged, in the words of [22], the third-level divide or digital divide outcomes or participation outcomes (at present), which is based on the social and cultural advantages derived from access to and use of digital tools [24], hence the importance of analysing the use of these tools to try to understand who has the greatest advantage [25].

In line with the above, Latin American countries, and Colombia in particular, despite having led the expansion of expenditure on ICTs, currently show dramatic differences in terms of both access to digital technologies and training in them [10,11,26]. For this reason, in order to reduce this gap, programmes and policies have been proposed for more than a decade to mitigate it [9,26], such as the regulation of telecommunications and internet services, providing online services, encouraging companies and individuals who want to undertake them and the promotion by the Ministry of Information Technologies and Communities (MinTIC) of an ICT Plan 2018–2022 called “*El futuro digital es de todos*” (“The digital future belongs to all”) that marks the momentum of connectivity and digital transformation of the Colombian territory in order to reduce the existing digital divide and to promote coverage and access to the Internet throughout the territory, among other aspects. For all these reasons, as the Institute of Education for Higher Education in Latin America and the Caribbean shows, the situation in Latin America is critically complex [26], as it faces unresolved challenges such as inequities in access to the system, and in the increases or decreases in public funding [15].

### 2.2. Inequalities in the Use (Know-How) of Digital Tools in the COVID-19 Era

As [15] points out, during this pandemic, the effectiveness of the use of these strategic resources in Higher Education for learning synchronous and asynchronous content has become feasible, turning them into competitive elements. In this sense, studies on the use of digital tools in education have experienced a considerable growth in recent years [23,27]. In the current situation, its exponential growth makes it necessary to analyse the changes that have taken place, the deepening of its nature, its diversification, its purposes, its functionality, the stakeholders involved in communication, the feelings and satisfaction it generated among its users, how competent they consider themselves to be, or the importance attributed to the activities performed with mobile devices [18]. This is followed by a brief review of the background and justification of studies focusing on the knowledge of digital tools in education; secondly, the use of measurement instruments for their study along the lines of the one explained here; the background to the validation of these instruments at this COVID-19 stage; and finally, what previous research on the existing digital divide has to offer, all of which justifies the need for the present study.

Firstly, previous studies on the use (know-how) show a low use of these digital tools for educational purposes by non-university students, with gender differences in the use of social networks being found [27]. As far as purposes are concerned, evidence from studies conducted in several different countries refers to a drastic change in recent years, switching from an interest that exclusively focused on leisure to a more intensive focus on solving everyday educational and interpersonal communication needs [18].

Secondly, the instruments used in recent studies on this topic are very diverse, ranging from the use of concrete non-standardised activities, programmes and instructional strategies such as gamification [28], either with writing and/or with specific tools such as e-quizzes for formative assessment, among others [28,29,30,31], specific questionnaires and self-reports, such as those dedicated to excessive and problematic internet use [32], the use of these tools as a means of peer harassment [33], to the use of various validated instruments [4,34]. For example, recent research has used DTS-type instruments such as the one conducted by [35], where 1374 American adults were surveyed in the first days of April 2020 on the use of various digital tools, the result of a much larger research project where the use of 983 Italian adults and 1350 Swiss adults was analysed [36] in addition to the above. In addition, other research has been specifically designed to examine teachers’ use of such web applications [37,38,39]. In relation to the validation of instruments during the pandemic, recent publications have multiplied greatly, examining different psychological variables both generally and in specific contexts [40,41,42,43], such as the emotional and psychological impact [44,45,46], mental health [47,48], isolation and social distance measures [49,50], changes in sexual behaviour [51], how it is affecting foreign students in other countries such as China [52], as well as the effects that the closure of educational institutions due to confinement have caused on mental health [53,54] in specific countries such as China [55], Italy [56] and Japan. However, one of the limitations observed that justifies this present study is the scarcity of validated instruments focused on the analysis of digital competences, and even less so in the field of Spanish, which also justifies this research.

Additionally, thirdly, recent studies report that the digital divide has diverse origins. On the one hand, studies describe the gap as a determining factor of health [9,57] as a function of personality traits [58], gender [19,58,59,60,61], culture [58,60,62], social class [12,17,19,58,60], geographical location [17,58,63], availability of technological means [9,37]. These studies report progress but identify important gaps. For example, they were conducted in cultural environments far away from the Latin American context, with greater availability of technological means and support such as advanced educational resources.

## 3. The Current Study

### 3.1. Research Questions

The research questions of this study are:

Q1 What visions do Latin American university students have of digital inequalities during the COVID-19 pandemic confinement and what role do demographic variables such as gender, socio-economic status (SSE), or employment status play in these visions?
Q2 What causal and mediating role do digital and demographic variables play in digital inequalities among Latin American university students during the period of confinement enacted to reduce the spread of COVID-19?

### 3.2. Objective and Hypothesis

In line with the previous research questions, the objective is to clarify whether or not there are digital inequalities in access or competence by analysing the use of eighteen groups of digital tools by young Latin American university students during the COVID-19 pandemic. This instrument will allow the determination of perceived differential patterns of frequency and duration of use. This will in turn contribute to the understanding of the third-level divide or digital divide outcomes or participation outcomes (at present), which is based on the social and cultural benefits derived from access to and use of digital tools. Therefore, a reliable and valid instrument needs to be developed and implemented, and mediational causal data need to be provided. This is embodied in the following assumptions concerning the digital divide:

**Hypothesis** **1** **(H1).** *Digital inequalities in access to and use of the eighteen groups of tools under analysis are found, advancing the understanding of the third-level divide.*

**Hypothesis** **2** **(H2).** *Differential patterns are found in terms of frequency and duration of use of selected tools based on gender, socio-economic status, and employment status, to the detriment of men, lower socio-economic levels and those who do not work.*

**Hypothesis** **3** **(H3).** *Frequency and duration of use do not mediate digital inequalities when considering stratum and employment status, nor do they moderate gender, but they do when assessing the purpose of use and the recipients of communication.*

## 4. Materials and Methods

### 4.1. Participants

In April and May 2020, during the first general confinement of the pandemic caused by the coronavirus disease, an online questionnaire was applied through simple random sampling to 5676 university students from a higher university institution on the Colombian Caribbean coast, representing around 40% of all students, with a mean age of 21.3 years, standard deviation of 4.6, 1481 men (51%) and 1401 women (49%), aged between 17 and 60, replying to the questionnaire in its entirety. According to the National Planning Department in Colombia, the participants belonged to all social strata, 90.5% from strata 1–3, the lowest, and only 9.5% from strata 4–6 (medium and high); in line with the majority distribution of middle and lower strata in the general Latin American population, subjects were from the different university degrees, majors, departments, and degree programmes (about 160 different subject types) and were representative of the very popular type of students trained in Latin American universities, with high-quality accreditation (see Table 1). Demographic data were verified and checked. They all agreed to participate voluntarily by giving informed consent online. The study was authorised by the University’s Ethics Committee.

About demographic data, 51.4% of participants were male compared to 48.6% who were female (*p* = 0.016). A total of 26.2% belonged to the first stratum, 37% to the second, 27.1% to the third, 8.3% to the fourth, 1% to the fifth and the rest to the sixth. In most strata, a higher proportion of males than females is found, excepting the second and fifth strata (*p* = 0.016). Similarly, 74.3% say they do not work, 14.2% say they work full-time and 11.5% say they work part-time (*p* = 0.001). In this case, there is a higher proportion of men than women among those who work part-time and full-time, and not among those who say they do not work (*p* = 0.001).

### 4.2. Instrument

An ad hoc online questionnaire, the Digital Technologies Survey (DTS) (see Supplementary Material File S1), is designed and applied through SurveyMonkey to examine the use of tools by eighteen groups of university students in different subjects of different degree courses. These technologies are grouped according to the area of use in four categories: educational: (i) blogs (Blogger, WordPress), (ii) wiki (Wikispaces, Mediawiki…), (iii) online word processing (Google Document…), (iv) online presentations (Prezi, SlideShare, Google Presentations…), (v) cloud storage (Drive, OneDrive, Dropbox…), (vi) online survey development (Forms Office, Google Forms, SurveyMonkey…), (vii) online response (Kahoot, Socrative, Poll Everywhere, Polldaddy…), (viii) online interactive notes (Pinterest, Lino It, Padlet…), (ix) recording (CamStudio, Screencast-O-Matic, Camtasia…), (x) video conferencing (Skype, FaceTime, Hangouts…); social: (xi) synchronous communication (WhatsApp, Telegram…), (xii) social networking (Facebook…), (xiii) image sharing (Instagram, Flickr, Picasa…), (xiv) microblogging (Twitter); fun: (xv) online series and film viewing (Netflix, HBO, Amazon Prime…) and (xvi) video (YouTube, Vimeo…); and professional: (xvii) academic/research social networking (Academia, ResearchGate…) and (xviii) professional social networking (LinkedIn).

The DTS is made up of several sections for each of the eighteen groups of digital tools: demographic data, frequency of use, duration of use, purpose of use, target audience, feelings, ability or competence to perform activities and importance of performing activities with mobile devices such as Smartphone or Tablet. (*i*) *Presentation of the study and socio-demographic data:* requesting voluntary participation and informed consent, after which they filled in their demographic data: date of birth, age, gender, whether they combine their studies with another job, family social stratum, current career, faculty and department. Independently verified demographic data. (*ii*) *Frequency of use:* the extent to which they had used those tools in their university studies, (*iii*) *Duration* of use, or time spent using the tools, on a 5-point Likert scale (less than one hour, between 1 and 3 h, between 3 and 6 h, between 6 and 9 h and more than 9 h). (*iv*) *Feelings* about use, on a 5-point Likert scale (not at all fun/pleasurable, not very fun/pleasurable, moderately fun/pleasurable, very fun/pleasurable, extremely fun/pleasurable). (*v*) *Ability or competence* to carry out specific activities with the selected digital tools, on a 5-point Likert scale (not at all competent, not very competent, moderately competent, very competent, extremely competent). (*vi*) *Importance of being competent* in performing a range of activities using mobile devices.

### 4.3. Procedure

**Figure 1:**
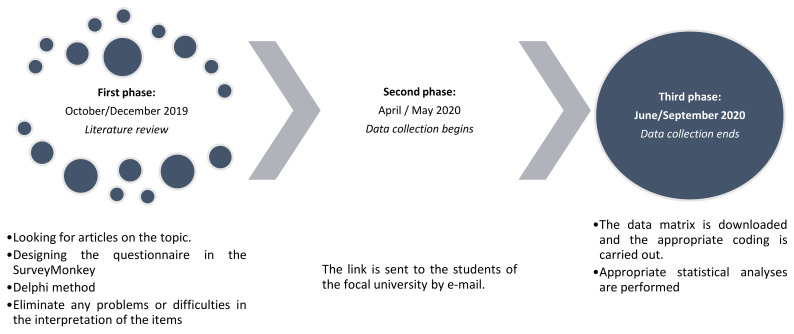
Study timeline.

As Figure 1 shows, first, instruments from previous international studies on the use of digital technologies are reviewed and analysed in order to articulate the variables analysed in the ad hoc questionnaire. After designing the questionnaire in the SurveyMonkey online software (Momentive—SurveyMonkey Europe UC, Dublin, Ireland), and following the Delphi method, it is sent to professionals in the field of specialisation, in order to verify its functionality and operability, and to eliminate any problems or difficulties in the interpretation of the items. Once modified, the link is sent to the students of the focal university by e-mail. Upon expiration of the open time set, the link is closed, the data matrix is downloaded, the appropriate coding is carried out, outliers are removed, and the appropriate statistical analyses are performed.

Firstly, descriptive, outlier, confirmation of the normality of the variables (skewness, kurtosis), cross-table, bivariate and multivariate analyses are carried out. The instrument was then validated. First, through exploratory factor analysis, EFA, with 50% of the sample, using maximum likelihood extraction, we conducted observation of the sedimentation plot and direct oblimin rotation. Kaiser–Meyer–Olkin (KMO) tests of sampling adequacy Bartlett’s test of sphericity and the χ^2^ goodness-of-fit test are calculated. Then, an internal consistency analysis of the items, Cronbach’s alphas, is performed. All these descriptive, consistency and EFA (construct validity) analyses are carried out using SPSS version 26. In contrast, the calculation of composite reliability CR (McDonald’s omega), extracted variance average EVA (convergent validity CV), square root of EVA, or CV (discriminant validity DV) is performed in Excel from the latent variable pattern matrices. Confirmatory factorial analysis (CFA) is then carried out in AMOS version 26 with the other 50% of the total sample, obtaining different re-specifications of the model, comparing the NFI (Normed Fix Index), TLI (Tucker–Lewis Index) and CFI (Comparative Fit Index), indices that must be above 0.90, and the RMSEA (Root Mean Square Error of Approximation), which must be below 0.08. Given that this is a very large sample, representative of the student body of a typical Latin American university with high-quality institutional accreditation and very diverse (both in terms of type of students and faculty, with multiple nationalities), the analyses are adequate, representative and generalisable.

## 5. Results

Once the instrument was validated, we performed the multivariate analysis using various GLM general linear models considering gender, stratum and employment status of the students as grouping variables, in order to answer the research question, using SPSS version 26. Finally, causal analyses were carried out to study the mediating role of the key variables taken into account, using the PROCESS macro version 3. 5 of Hayes (2012–2020) with SPSS version 26, which uses the bootstrapping technique through simulation of 10,000 samples, identifying intervals of effects that are considered significant if they do not include 0 in them. The direct effects derived from the causal variables (X) on the outcomes (Y) (c’ coefficients), and the indirect effects produced by the mediating variables (M) can be calculated, along with both the effects of X on M (ai coefficients) and the effects of M on Y (bi coefficients), as well as the total effects (c).

### 5.1. DTS Validation

#### 5.1.1. Exploratory Factor Analysis (EFA)

Analyses for the EFA calculations for construct validity provide evidence of the adequacy of psychometric properties. On the one hand, the EFA provides construct validity of three latent variables as the best solution. The Kaiser–Meyer–Olkin (KMO) test gives a very high coefficient of 0.938, indicating good sampling adequacy, and Bartlett’s test of sphericity was highly significant at 0.001. The goodness-of-fit test is highly significant, with a χ^2^ with *p* < 0.001. It is true that the measures of use of the eighteen types of tools, with whom they communicate, and the purpose of the tools are dichotomous variables, so it is not possible to include them in these analyses, although a Cronbach’s alpha internal consistency of 0.777 for use or 0.668 for with whom they communicate is obtained. The first latent variable, perceived importance and competence in digital activities, is made up of indicators derived from the assessment of the importance of activities with mobile devices together with feeling skilled and competent in carrying out digital activities; it explains 17.5% of the total variance. McDonald’s composite reliability CR/omega amounts to 0.96 (must be equal to or greater than 0.70); the extracted variance average EVA or convergent validity CV is 0.512 (must be equal to or greater than 0.50); the discriminant validity DV or square root of the convergent validity CV is 0.716 (must be greater than the correlations between the latent variables: r_12_ = 0.233; r_13_ = −0.09; r_23_ = −0.214). The second latent variable, pleasant feelings derived from the use of digital tools, accounts for 11% of the total variance explained, and includes indicators relating to whether the use of virtual tools is considered fun or pleasurable. McDonald’s compound reliability/omega is 0.93 (must be equal to or greater than 0.70); the extracted variance average EVA or convergent validity CV is 0.505 (must be equal to or greater than 0.50); the discriminant validity DV or square root of the convergent validity CV is 0.71 (must be greater than the correlations between the latent variables: r_12_ = 0.233; r_13_ = −0.09; r_23_ = −0.214). Finally, the third latent variable, duration or time spent using digital tools, accounts for 6% of the total variance explained. The compound reliability of CR is 0.84; the extracted variance average EVA or convergent validity CV is 0.324 (should be at least 0.50), the discrimination validity DV or square root of the convergent validity CV is 0.57 (higher than the inter-correlations between latent variables: r_12_ = 0.233; r_13_ = −0.09; r_23_ = −0.214).

#### 5.1.2. Confirmatory Factorial Analysis (CFA)

**Figure 2:**
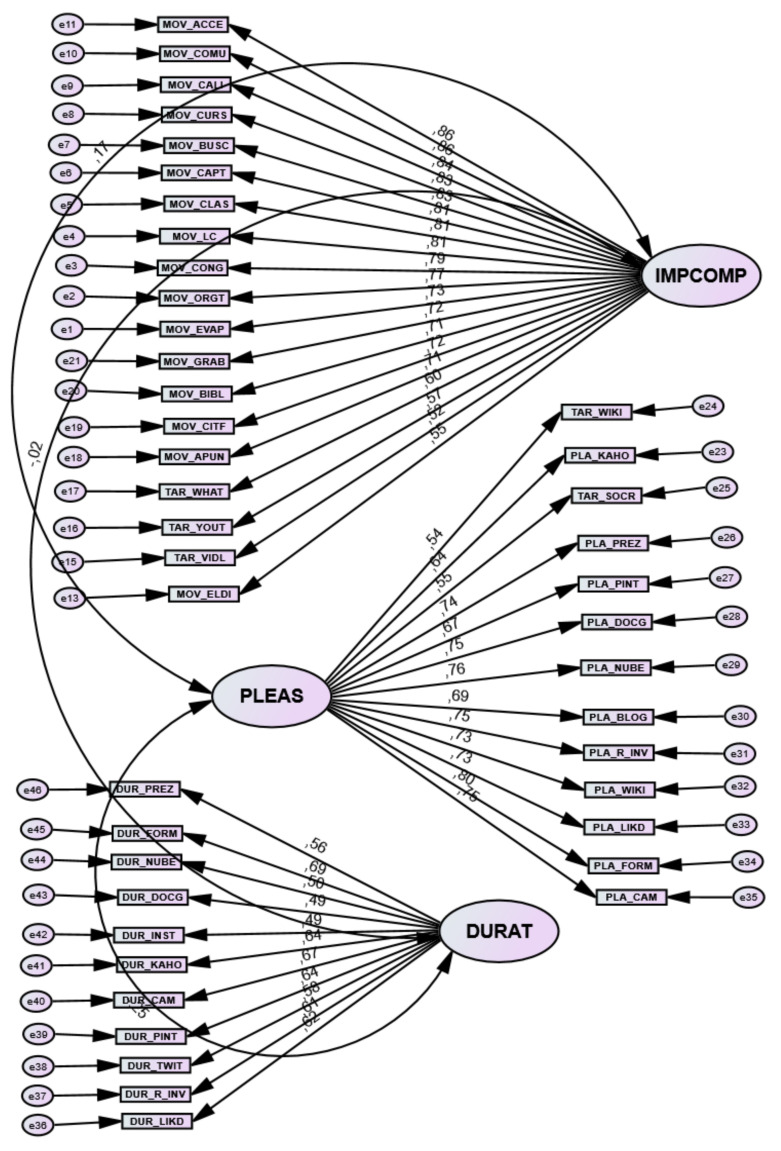
Three-factor measurement model of CFA confirmatory factor analysis of the DTS: IMPCOMP Importance-Competence; PLEAS Pleasurable; DURAT Duration or time.

Once the factors have been identified using the EFA, the measurement model is submitted for confirmation. After re-specifying the different models, the resulting final model gives NFI = 0.908, TLI = 0.907 and CFI = 0.917 (these indices should be above 0.90) and an RMSEA = 0.054 (should be below 0.08). This confirms the model’s adequacy. Considering the results of internal consistency reliability, composite reliability, content, construct, convergent and discriminant validity, together with the adequacy of the goodness of fit in the measurement model (CFA), it can be affirmed that the instrument used is reliable and valid (see Figure 2).

### 5.2. Univariate and Multivariate Analysis

To answer the first research question Q1. What visions do Latin American university students have of digital inequalities during the COVID-19 pandemic confinement and what role do demographic variables such as gender, socio-economic status, or employment status play in these visions? Univariate and multivariate analyses (General Linear Models) are carried out.

#### 5.2.1. Univariate Analyses

As Table 2 shows, in terms of frequency, according to gender, statistically significant differences are shown in blogs, wikis, recording tools, cloud storage, online response, online interactive notes, image sharing tools, microblogging and professional social networking. On the other hand, in the stratum variable, differences are shown in wikis, video, online series and film viewing tools, microblogging and in the professional social network. Finally, in relation to the employment status during the period of application of the instrument, there are differences in blogs, videoconferencing tools, online presentations, recording tools, online response academic/research social networks, and professional social networks. In relation to the duration of use, according to gender, statistically significant differences are found in blogs, wikis, synchronous communication tools, online presentation tools, recording, online survey making, online response, online interactive notes, microblogging, in academic/research social networks and in the professional social network. In terms of stratum, they are found in blogs, video, online series and movie viewing tools, online word processing tools, online survey tools, online interactive notes, image sharing applications, microblogging and professional social networking. Finally, with regard to the employment status during the period of application of the instrument, differences are found in synchronous communication tools, video, the viewing of online series and films, online response, image sharing, academic/research social networks and the professional social network.

#### 5.2.2. Multivariate Analyses (GLM)

Three types of multivariate analyses of variance are carried out using the general linear model (GLM) with fixed factors (i) gender, (ii) socio-economic stratum and (iii) employment status at the time of the questionnaire and dependent factors being measures of perceptions of frequency of use and duration of use of the eighteen groups of digital tools.

##### Gender

When considering gender as a fixed factor, multivariate contrasts show statistically significant differences with a median effect size (Wilks’s Lambda = 0.752, F_(1828.00, 127)_ = 4.738, *p* ≤ 0.001; η^2^ = 0.248).

As for the inter-subject tests, as presented in Table 3, statistically significant differential patterns with small effect sizes in favour of women are shown in variables related to the frequency of use of the educational and professional digital technologies such as LinkedIn, of digital video recording tools such as Camstudio, Screencast O Matic, Camtasia and in taking online surveys such as Kahoot, Socrative, Poll Everywhere and Polldaddy. On the other hand, men report a higher frequency of use of educational and social tools for creating interactive online notes such as Pinterest, Lino It, Padlet and for viewing or sharing images on Instagram or Flickr (see Table 3).

Similarly, there are statistically significant differences in the duration of these groups of tools. In this sense, it is found that women spend more time on synchronous communication tools such as WhatsApp or Telegram than men (e.g., M_SyncCommToolsHerramComunicSincroWomen_ = 3.50 vs. M_SyncCommToolsHerramComunicSincroMen_ = 3.62; *p* = 0.042; η^2^ = 0.002), who on the other hand favour video viewing on tools such as YouTube and Vimeo (e.g., M_VideoToolsMen_ = 3.12 vs. M_VideoToolsWomen_ = 2.82; *p* = ≤0.001; η^2^ = 0.013).

##### Stratum

When considering stratum as a fixed factor, multivariate tests show statistically significant differences with a small effect size (Wilks’s Lambda = 0.693, F _(9125, 630)_ = 1.105, *p* = 0.039; η^2^ = 0.071).

In terms of tests of inter-subject effects, statistically significant differences with small effect sizes are shown for variables related to the frequency of use and duration for the eighteen groups of digital tools examined.

In line with the above, on the one hand, in terms of frequency, educational tools are used more frequently by students in stratum 6, except for interactive notes that are used more frequently by those in stratum 1. However, in relation to social tools, specifically Twitter, surprisingly, a slight reduction in the frequency of use as the stratum increases is observed. In this regard, it is plausible that the group of paid tools for watching online series and films such as Netflix, HBO and Amazon Prime is higher in stratum 6 than in the rest, which is evidence, once again, of the well-known digital divide. However, it is striking that stratum 5 has the lowest score in all the tools examined, followed by stratum 4 and then the rest of the strata in different order according to the tool group examined (see Table 4).

On the other hand, in relation to duration, it is surprising that the duration of use of social tools, specifically instant messaging, increases as the stratum increases. In this respect, differential patterns are found depending on the socio-economic stratum and the selected tool group. In this sense, the same pattern is observed for synchronous communication tools (WhatsApp, Telegram…) and paid online series and films (Netflix, HBO, Amazon Prime…), with an increase in the duration of use as the stratum increases (see Table 5).

The post hoc comparisons that yielded statistically significant differences are only found in the frequency of Twitter use when comparing stratum 1 with the stratum 3 variable (M_S1_ = 1.34 vs. M_S3_ = 1.24; *p* = 0.042) and in YouTube duration between stratum 4 and stratum 1 and 2 (M_S4_ = 3.36 vs. M_S1_ = 2.88; *p* = 0.013; vs. M_S2_ = 2.91; *p* = 0.018).

##### Employment Status

When considering employment status during the period of application of the instrument as a fixed factor, multivariate contrasts show statistically significant differences with a median effect size (Wilks’s Lambda = 0.805, F_(3656, 252)_ = 1.663, *p* ≤ 0.000; η^2^ = 0.103). On the other hand, in relation to the inter-subject tests, statistically significant differences with small effect sizes are found in variables related to the frequency of use of sharing wiki content, preparing online presentations and carrying out online surveys in favour of students who were working full-time at the time the questionnaire was applied, except in the case of the variable of communicating professionally (LinkedIn), where a higher frequency of use is evident among those who were unemployed (see Table 6).

On the other hand, in terms of duration, statistically significant differences are found in the sharing wiki content, synchronous communication, conducting online surveys and communicating professionally variables (see Table 7).

Finally, when post hoc testing is performed, several differential patterns are found in terms of frequency of use and duration for the selected groups of tools.

In this sense, on the one hand, in terms of frequency of use, the first pattern materialises in a higher use of wiki content sharing tools by students who are working full time than by those who are working part-time (M_FT_ = 1.48 vs. M_PT_ = 1.34; *p* = 0.011). Similarly, there is a greater use of online survey tools (Kahoot, Socrative…) among those who do not work and those who work full-time (M_NO_ = 1.29 vs. M_FT_ = 1.41; *p* ≤ 0.001). Similarly, higher use of the professional social network (LinkedIn) is found among those who do not work and those who work full-time (M_NO_ = 1.56 vs. M_FT_ = 1.37; *p* ≤ 0.001).

On the other hand, in relation to the duration of use of the groups of tools, a longer duration of use is observed among students who are working full-time than those who are part-time when it comes to sharing content on wikis (M_FT_ = 3.31 vs. M_PT_ = 2.82; *p* = 0.040). Similarly, students who do not work and those who work part-time also use synchronous communication tools (WhatsApp, Telegram, etc.) more frequently (M_NO_ = 3.59 vs. M_PT_ = 3.31; *p* = 0.023). Finally, longer duration of the use of online survey tools (Kahoot, Socrative…) is found among students who are working full-time than those who are not working or those who are working part-time (M_FT_ = 3.26 vs. M_NO_ = 2.71; *p* ≤ 0.001; vs. M_PT_ = 2.77; *p* = 0.032).

### 5.3. Mediational Causal Models

**Figure 3:**
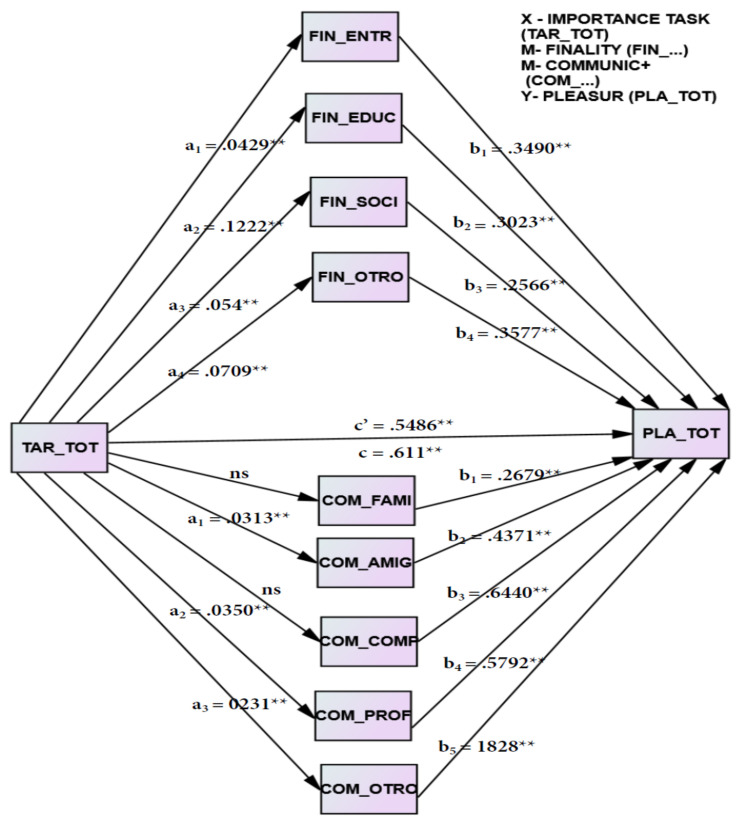
Mediation model of how the importance of digital task influences the satisfaction and pleasure of the use of digital technologies via finality of the tools and communication with those tools. Causal mediational analysis X → M → X, being the indirect effects of X (importance of the digital task) on outcome Y (feelings of digital activities) through the variable M (purpose and communication), a_i_ and b_i_ and the direct effects c′ of X over Y, with the total effects c. **: *p* < 0.01.

In order to answer the second research question (Q2. What causal and mediating role do digital and demographic variables play in digital inequalities among Latin American university students during the period of confinement enacted to reduce the spread of COVID-19?), different mediational causal analyses were implemented considering the importance of the digital activity undertaken as a predictor or causal variable (X), the feelings of satisfaction through use of the digital tools analysed as a predictor or outcome variable (Y), social stratum and employment status as mediating variables, and gender as a moderating variable. Both the moderating variable gender and the mediating variables social stratum and employment status do not give relevant results in this causal relationship, so no diagram is included. When the purpose of digital tools or with whom digital tools are communicated with are included as mediating variables, and statistically significant results are obtained, adding value to the understanding of the causal role of a relationship between the importance attached to the digital activity in personal satisfaction with its use, mediated by the purpose and at whom communication with these tools is aimed (see Figure 3).

#### 5.3.1. Causal Relevance of Purpose of Digital Activities as a Mediator

In particular, the nuance they introduce, both the purpose of using digital tools and who communicates with them, must be considered for a correct understanding of the equation. The importance of the digital activity (X_ACT_) directly predicts (c′) satisfaction with its use (Y_SAT_) with a highly significant coefficient (c′ = 0.5486, *p* > 0.001); it also indirectly predicts the mediating variable purpose (M_PUR_) in a statistically significant way, both for entertainment (a_1_ = 0.0429, *p* < 0.0036), education (a_2_ = 0.1222, *p* < 0.001), social (a_3_ = 0.054, *p* < 0.0004), and other (a_4_ = 0.0709, *p* < 0.001). The mediating variables purpose of the use of digital tools (M_PUR_) indirectly predict satisfaction with digital tools (Y_SAT_), each for entertainment (b_1_ = 0.3490, *p* < 0.001), education (b_2_ = 0.3023, *p* < 0.001), social (b_3_ = 0.2566, *p* < 0.001) and other purposes (b_4_ = 0.3577, *p* < 0.001). The bootstrapping intervals obtained to identify the coefficients and their statistical significance show that the total effects of X on Y have a coefficient c = 0.611, so the relevance of the indirect effects of the importance of the digital activity (X_ACT_) through the mediating variables (M_PUR_) on the satisfaction variable with digital activities or outcome (Y_SAT_) is relevant and should be considered for a correct understanding of the use of tools, playing a role from highest to lowest, that of other purposes, followed by entertainment, education, and finally the social purpose.

#### 5.3.2. Causal Relevance of Who Communicates with Digital Activities as Mediator

This is similar for the mediating variables of who communicates using virtual tools (M_COM_), which mediate between the importance of the digital activity (X_ACT_) as a predictor or causal variable in the satisfaction variable with digital activities or outcome (Y_SAT_). On the one hand, the importance of the digital activity (X_ACT_) has an indirect effect on the mediating variable with whom they communicate using virtual tools (M_COM_), with friends (a_1_ = 0.0313, *p* < 0.001), teachers (a_2_ = 0.0350, *p* < 0.001) and with others (a_3_ = 0231, *p* < 0.001); it is not statistically significant with family members or peers. On the other hand, in the second part of the equation, the mediating variables with whom they communicate using virtual tools (M_COM_) predict in a relevant and indirect way the satisfaction variable with digital activities or outcome (Y_SAT_), with family (b_1_ = 0.2679, *p* < 0.001), friends (b_2_ = 0.4371, *p* < 0.001), peers (b_3_ = 0.6440, *p* < 0.001), teachers (b_4_ = 0.5792, *p* < 0.001) or others (b_5_ = 1828, *p* < 0.001). The direct effects of X on Y, c′ = 0.5165 are statistically significant, the total effects being as in the mediation equation above. After bootstrapping, interval analysis gives statistically significant results when comparing the indirect effects of family vs. friends, family vs. teachers, friends vs. others and teachers vs. others, with the other comparisons not being statistically significant. From highest to lowest, communication with peers through digital tools, with teachers, with friends, with family and finally with others, play a significant part in the predictive role, as can be seen from the magnitude of the coefficients.

## 6. Discussion

In 2020, the importance that the use of digital tools acquired globally for the development of online education marked a turning point [64]. To this end, the COVID-19 pandemic has resulted in an unprecedented crisis in the higher education space, making social, cultural, economic and—of course—digital inequalities more visible [1,2,4,5,6,7]. In recent months, Colombian education systems have been forced to deploy learning modalities on virtual platforms through various formats (synchronous or asynchronous) and platforms with or without access to digital technologies [26]. For this reason, according to Junco (2013) in this article, it is a priority to glimpse the visions that young Latin American university students have of digital inequalities based on gender, socioeconomic status, and employment status, during the period of confinement decreed to reduce the spread of the COVID-19 disease. In line with the above, the results have shown the existence of statistically significant differences when considering gender in the frequency of use of the educational tools examined, in social technologies with a slight increase in favour of men and in professionals with an increase in women. Likewise, differences have also been glimpsed when socioeconomic status is considered as a grouping variable in the frequency and duration of use of educational, social and entertainment tools with a certain benefit, in general terms, in the highest strata. Similarly, statistically significant differences have been observed when considering employment status in the frequency and duration of educational, social, and professional technologies. The causal and mediating role of various demographic and usage variables should also be clarified.

In this sense, this research made a breakthrough in understanding the third-level divide by confirming the existence of social and cultural benefits derived from the use of the digital tools examined [21]. In this regard, as described in the Results Section, students from higher socio-economic strata have access to more digital resources and, consequently, have the possibility of making greater use of them than those from lower socio-economic strata. This is the case of digital applications under subscriptions such as Netflix, HBO or Amazon Prime [65]. However, this trend is also observed in the evolution of the use of the first to the fourth stratum on video applications that do not require a subscription and in tools to share images such as Instagram or Flickr or in synchronous communication technologies such as WhatsApp or Telegram, which in turn has a positive impact on them, as it reduces the psychological and emotional impact of COVID-19 confinement on university students [57] as it favours disconnection from the current epidemiological situation.

Given the context described above arising from current concerns about equity of access to computers and the Internet [61] and the large number of students using a variety of digital tools in higher education, this study analyses how the gender of university students might influence their visions of these tools in the learning context. As Correa, Hinsley and Zuniga pointed out in 2010, knowing and understanding these differences is the first step for teachers to design effective learning environments. In this regard, both use educational tools frequently. However, women also show frequent use of professional ones compared to men who make more frequent use of social ones. These results do not coincide with those obtained by García-Martín and García-Sanchez in 2015. On the other hand, in terms of socioeconomic level, it is observed that educational tools are, in general, used more frequently by students in stratum 6 than by those in stratum 1. In addition, the average time that young Latin Americans spend on social tools, specifically on instant messaging applications such as WhatsApp, Telegram… increases as the socioeconomic level increases. However, in the case of Twitter, a slight decrease is observed. Finally, regarding the employment situation, there is a glimpse of a greater use of instant messaging technologies such as WhatsApp and Telegram among students who do not work and those who work part-time, as well as increased use of LinkedIn among non-working and full-time students.

Furthermore, regarding to the educational and social implications, this study has shown that the variables of frequency and duration of use do not mediate digital inequalities when considering socio-economic status and employment status, nor do they moderate gender, but they do when considering the purpose of use and the recipients of the communication. Therefore, this study has confirmed that instant messaging communication via computers and mobile devices promotes social presence in students, with students exercising control over when and with whom they interact, generating numerous opportunities to integrate learning in Higher Education [66], thus advancing the understanding of the third level divide in terms of social and cultural benefits derived from the use of digital tools [21], making it possible to anticipate the undeniable risk of increasing inequality and to try to address the threat of the much-feared digital stratification in time.

## 7. Conclusions

This study makes various contributions around the objective regarding digital inequalities in access or competition through the analysis of the use of eighteen digital technologies grouped into four categories based on their function: educational, social, fun, and professional. In this sense, firstly, we provide empirical validation of an ad hoc online questionnaire useful in Latin American universities to detect the state of the art on the use (know-how) of digital tools in terms of their use, duration, purpose, communication recipients, feelings of satisfaction, and perception of competence with different educational activities [27]. Furthermore, it is possible to explore the differential patterns that these components provide for decision-making to improve the quality of educational assessment and intervention [67]. Secondly, it provides an analysis of differential patterns that may contribute to explaining the digital divide and inequalities that condition access to university education, predominantly virtual during this pandemic [23]. Thirdly, it explains the main factors related to digital tools and digital competence in university assessment and teaching, and which should be considered, introducing aids to strengthen the gaps and weaknesses of university students in digital competence due to gender, stratum, employment status, and others that should be considered in the future, such as cultural factors, rural or urban origin, or the department and faculty to which they are being trained, among others.

However, there are several limitations and future perspectives should focus on overcoming them. On the one hand, although the participating university students have similar characteristics to those in other Latin American universities, with international faculty from many countries, it seems relevant to extend the samples to other university institutions, and even to compare different cultural environments. It would be advisable to analyse causal factors, for example, the role of university teachers teaching and assessment methodologies in academic performance, or in the enhancement of key psychological variables such as academic self-efficiency or coping with difficulties, and how digital competence variables mediate and moderate this causal relationship. For example, media analyses are identifying a relevant role of duration, purpose, communication, satisfaction, competence, or the importance of digital technologies in the causal relationship between assessment systems and teaching methodologies in the different standard subjects of all grades in enhancing the belief in the ability to cope with educational challenges and academic achievements. It would be interesting to extend the analysis of the causal relationships between digital competence and different psychological and educational variables in academic results and in the enhancement of factors that have a direct impact on the improvement of the quality of university education. The role that the excessive or problematic use of these tools plays in the adaptation of students and the constraints they impose on their educational, personal, and social development is another interesting issue. Analyses show that measures such as Problematic Internet Use (PIU) should be considered [32], as these measures have an impact on the adaptive functioning and learning potential of university students, reducing their capacity for self-regulation and independent learning.

Moreover, the theoretical and practical implications of this study are diverse. On the one hand, assessments of competences and digital tools will identify training gaps and specific needs for action, e.g., in the implementation of the use of tools that facilitate autonomous and self-regulated learning, or that have a particular focus on specific subjects and fields (e.g., mathematics or literacy across disciplines) [68,69]. It will also enable the establishment of psychoeducational profiles for the design of innovative instructional methodologies such as flipped learning or blended learning [70]. In this sense, the development of gamification and its contribution and usefulness for the systematic implementation of the assessment and teaching of generic and specific competences of university students [29,30,31], or simulation-based learning [71] is relevant. The changes being introduced by the new normality will result in higher levels of demand in the use and command of digital tools, so that those who cannot access their possibilities, due to their vulnerability, will see their academic progress and subsequent social ascent hindered. Digital tools make possible other ways of assessing and teaching at university, where blended learning classes, gamification, and the use of MOOCs [67] will be a privileged way, together with other never imagined ways of fostering digital competences [72,73].

## Figures and Tables

**Table 1:** Sociodemographic data of the participants: genre, SSE and employment situation.

	Gender		Total
	Men	Women	
	1481 (51.4 %)	1401 (48.6%)	2882
Socioeconomic status—SSE (M = 2.22; SD = 99)			
SSE 1 (Low-Low)	399 (53%)	356 (47%)	755 (26%)
SSE 2 (Low)	513 (48%)	552 (52%)	1065 (37%)
SSE 3 (Medium-Low)	414 (53%)	368 (47%)	782 (27%)
SSE 4 (Medium)	136 (57%)	102 (43%)	238 (8%)
SSE 5 (Medium-High)	10 (34.5%)	19 (65.5%)	29 (1%)
SSE 6 (High)	9 (69%)	4 (31%)	13 (0.5%)
	Employment situation		
Does not work	1063 (50%)	1077 (50%)	2140 (74%)
Work part-time	200 (60%)	132 (40%)	332 (11.5%)
Work full time	218 (53%)	192 (47%)	410 (14%)
	Total		2882

**Table 2:** Pearson’s chi-squared test.

		Frequency			Duration		
		Gender	Stratum	Employed Status	Gender	Stratum	Employed Status
Educational	Blogs (Bloogger, WordPress…)	0.001		0.001	≤0.001	0.034	
	Wikis (Wikispaces, Mediawiki…)	≤0.001	0.001		≤0.001		
	Online word processing tools (Google Document…)					0.023	
	Online presentations (Prezi, SlideShare, Google Presentations…)			0.008	≤0.001		
	Cloud storage (Drive, OneDrive, Dropbox…)	0.001					
	Online survey development (Forms Office, Google Forms, SurveyMonkey…)				≤0.001	0.049	
	Online response (Kahoot, Socrative, Poll Everywhere, Polldaddy…)	0.009		0.009	≤0.001		0.008
	Online interactive notes (Pinterest, Lino It, Padlet…)	≤0.001			≤0.001	≤0.001	
	Recording tools (CamStudio, Screencast-O-Matic, Camtasia…)	≤0.001		0.012	≤0.001		
	Videoconferencing tools (Skype, FaceTime, Hangouts…)			0.002			
Social	Synchronous communication tools (WhatsApp, Telegram…)				≤0.001		≤0.001
	Image sharing tools (Instagram, Flickr, Picasa…)	0.001				0.009	≤0.001
	Microblogging (Twitter)	0.019	0.004		0.004	0.018	
Fun	Online series and film viewing tools (Netflix, HBO, Amazon Prime…)		≤0.001			0.005	≤0.001
	Video (YouTube, Vimeo…)		0.002			0.017	0.020
Professional	Academic/research social networks Academia, (ResearchGate…)			0.036	0.025		≤0.001
	Professional social networking (LinkedIn)	≤0.001	0.001	0.001	≤0.001	0.021	≤0.001

Note: Only statistically significant data are shown.

**Table 3:** Frequency of use of digital technologies according to gender as a fixed factor.

	Digital Technologies/Gender	Men	Women	*p*	η^2^
Educational	Conduct online surveys (Kahoot, Socrative, Poll Everywhere, Polldaddy…)	1.28 (0.450)	1.34 (0.472)	0.010	0.003
	Create interactive notes online (Pinterest, Lino It, Padlet…)	1.38 (0.487)	1.29 (0.453)	≤0.001	0.010
	Record (Camstudio, Screencast O Matic, Camtasia…)	1.39 (0.488)	1.53 (0.499)	≤0.001	0.020
Social	View or share images (Instagram, Flickr, Picasa…)	1.10 (0.295)	1.07 (0.253)	<0.029	0.002
Professional	Communicate professionally (LinkedIn)	1.48 (0.500)	1.58 (0.494)	≤0.001	0.010

Note: Only statistically significant data are shown.

**Table 4:** Frequency of use according to the socioeconomic stratum (SES1 lower to SES6 higher) as a fixed factor.

	Digital Technologies/SES	SES1	SES2	SES3	SES4	SES5	SES6	*p*	η^2^
Educational	Blog (Blogger, WordPress…)	1.42 (0.495)	1.38 (0.486)	1.35 (0.478)	1.43 (0.497)	1.12 (0.332)	1.43 (0.535)	0.033	0.006
	Wiki (Wikispace, Mediawiki…)	1.45 (0.498)	1.42 (0.494)	1.36 (0.479)	1.40 (0.492)	1.29 (0.470)	1.71 (0.488)	0.015	0.007
	Videoconference (Skype, FaceTime, Hangouts)	1.12 (0.323)	1.10 (0.304)	1.06 (0.245)	1.07 (0.247)	1.00 (0.000)	1.29 (0.488)	0.007	0.008
	Online interactive notes (Pinterest, Lino It, Padlet)	1.38 (0.485)	1.36 (0.480)	1.29 (0.456)	1.29 (0.455)	1.06 (0.243)	1.14 (0.378)	0.003	0.009
Social	Microblogging (Twitter)	1.34 (0.474)	1.32 (0.468)	1.24 (0.428)	1.28 (0.449)	1.12 (0.332)	1.29 (0.488)	0.005	0.009
Fun	Video (YouTube, Vimeo…)	1.03 (0.165)	1.04 (0.186)	1.01 (0.116)	1.06 (0.237)	1.00 (0.000)	1.00 (0.000)	0.042	0.006
	Watching series and movies online (Netflix, HBO, Amazon Prime)	1.10 (0.301)	1.08 (0.269)	1.06 (0.231)	1.11 (0.309)	1.00 (0.000)	1.29 (0.488)	0.018	0.007

Note: Only statistically significant data are shown.

**Table 5:** Duration according to the socioeconomic stratum (SES1 lower to SES6 higher) as a fixed factor.

	Digital Technologies/SES	SES1	SES2	SES3	SES4	SES5	SES6	*p*	η^2^
Educational	Share wiki content (Wikispace, Mediawiki…)	3.21 (2.105)	3.08 (2.030)	2.81 (1.973)	3.27 2.061)	3.12 (1.996)	2.86 (2.410)	0.031	0.006
Social	Synchronous communication (WhatsApp, Telegram…)	3.43 (1.319)	3.55 (1.342)	3.64 (1.308)	3.72 (1.324)	3.94 (1.519)	4.00 (4.155)	0.045	0.006
	Share images (Instagram, Flickr, Picasa…)	2.92 (1.500)	2.96 (1.466)	3.04 (1.458)	3.36 (1.458)	3.24 (1.437)	2.86 (1.345)	0.026	0.007
Fun	Watch video (YouTube, Vimeo…)	2.88 (1.295)	2.91 (1.321)	3.03 (1.326)	3.33 (1.280)	3.06 (1.326)	3.00 (1.155)	0.004	0.009
	Watch series and movies online (Netflix, HBO, Amazon Prime)	3.02 (1.430)	3.02 (1.352)	3.14 (1.339)	3.36 (1.360)	3.35 (1.412)	3.43 (1.397)	0.047	0.006

Note: Only statistically significant data are shown.

**Table 6:** Frequency of use according to the employment situation (no work, part-time PT, full time FT) as a fixed factor.

	Digital Technologies/Employment Situation	NO	PT	FT	*p*	η^2^
Educational	Share wiki content (Wikispace, Mediawiki…)	1.41 (0.492)	1.34 (0.476)	1.48 (0.501)	0.009	0.005
	Prepare online slides (Prezi, SlideShare, Google Slides…)	1.07 (0.254)	1.10 (0.299)	1.11 (0.317)	0.028	0.004
	Conduct online surveys (Kahoot, Socrative, Poll Everywhere, Polldaddy…)	1.29 (0.452)	1.35 (0.477)	1.41 (0.493)	≤0.001	0.009
Professional	Communicate professionally (LinkedIn)	1.56 (0.497)	1.49 (0.501)	1.37 (0.485)	≤0.001	0.016

Note: Only statistically significant data are shown.

**Table 7:** Duration of use according to the employment situation (no work, part time PT, full time FT) as a fixed factor.

	Digital Technologies/Employment Situation	No	PT	FT	*p*	η^2^
Educational	Share Wiki content (Wikispace, Mediawiki…)	3.04 (2.033)	2.82 (1.929)	3.31 (2.155)	0.035	0.003
	Prepare online surveys (Kahoot, Socrative, Poll Everywhere, Polldaddy…)	2.71 (1.933)	2.77 (1.891)	3.26 (2.152)	≤0.001	0.009
Social	Synchronous communication (WhatsApp, Telegram…)	3.59 (1.321)	3.31 (1.353)	3.58 (1.341)	0.022	0.004
Professional	Communicate professionally (LinkedIn)	3.43 (2.200)	3.25 (2.090)	3.08 (2.107)	0.039	0.003

Note: Only statistically significant data are shown.

## Data Availability

Any information on the instrument (Supplementary Material File S1) or the original data will be provided by the authors if required.

## Supplementary Materials

The following are available online at https://www.mdpi.com/article/10.3390/ijerph19063358/s1, File S1: Digital Technologies Survey (DTS).
