# Supplementary material for: The Digital Divide of Know-How and Use of Digital Technologies in Higher Education: The Case of a College in Latin America in the COVID-19 Era
Source: Int J Environ Res Public Health. 2022 Mar 12;19(6):3358. doi: 10.3390/ijerph19063358 (PMC8954789; doi:10.3390/ijerph19063358)
Supplement: Supplementary file 1 [file ijerph-19-03358-s001.zip › ijerph-1577152-supplementary.pdf]

International Journal of
Environmental Research and Public Health

Digital Technologies Survey (DTS)

PRESENTATION OF THE STUDY

This is an investigation that aims to evaluate the use made of eighteen groups of digital tools during their university studies. That is why we ask for your valuable participation in the following survey. The estimated time of effective work, to adequately answer the survey, is 15 minutes. There are no right or wrong answers, just common situations and different practices. The best answer is the one that honestly reflects your reality and point of view.

Your answers are confidential and only processed for global statistical purposes, in accordance with the ethical and deontological standards applicable to all scientific research.

We thank you for your generous and necessary participation without which this research would not be possible.

Answer all questions taking enough time, and click send so that we can count on your effective participation.

Do I confirm that I have been informed and that I wish to participate in the research? Yes / No

SOCIO-DEMOGRAPHIC DATA

1- Data of birth	2- Age	3-Gender	4. Family social stratum
5- Current carrer	6-Faculty	7- Departament	8- Current career

DIGITAL TECHNOLOGIES ON THE UNIVERSITY SCENARIO

FREQUENCY OF USE

	To what extent have you used in your university studies ...?					
EDUCATIONAL	blogs (Blogger, Wordpress...)	*Never*	*Almost never*	*Sometimes*	*Almost always*	*Always*
	wikis (Wikispaces, Mediawiki...)	*Never*	*Almost never*	*Sometimes*	*Almost always*	*Always*
	online word processing (Google Document...)	*Never*	*Almost never*	*Sometimes*	*Almost always*	*Always*
	online Presentations (Prezi, SlideShare, Google Presentations...)	*Never*	*Almost never*	*Sometimes*	*Almost always*	*Always*
	cloud storage (Drive, OneDrive, Dropbox...)	*Never*	*Almost never*	*Sometimes*	*Almost always*	*Always*
	online survey development (Forms Office, Google Forms, SurveyMonkey...)	*Never*	*Almost never*	*Sometimes*	*Almost always*	*Always*
	online response (Kahoot, Socrative, Poll Everywhere, Polldaddy...)	*Never*	*Almost never*	*Sometimes*	*Almost always*	*Always*
	online interactive notes (Pinterest, Lino It, Padlet...)	*Never*	*Almost never*	*Sometimes*	*Almost always*	*Always*
	recording (CamStudio, Screencast-O-Matic, Camtasia...)	*Never*	*Almost never*	*Sometimes*	*Almost always*	*Always*
	video conferencing (Skype, FaceTime, Hangouts...)	*Never*	*Almost never*	*Sometimes*	*Almost always*	*Always*
SOCIAL	synchronous communication (WhatsApp, Telegram...)	*Never*	*Almost never*	*Sometimes*	*Almost always*	*Always*
	social networking (Facebook...)	*Never*	*Almost never*	*Sometimes*	*Almost always*	*Always*
	image sharing (Instagram, Flickr, Picasa...)	*Never*	*Almost never*	*Sometimes*	*Almost always*	*Always*
	microblogging (Twitter)	*Never*	*Almost never*	*Sometimes*	*Almost always*	*Always*
FUN	online series and film viewing (Netflix, HBO, Amazon Prime...)	*Never*	*Almost never*	*Sometimes*	*Almost always*	*Always*
	video (YouTube, Vimeo...)	*Never*	*Almost never*	*Sometimes*	*Almost always*	*Always*
PROFESSIONAL	academic/research social networking (Academia, ResearchGate...)	*Never*	*Almost never*	*Sometimes*	*Almost always*	*Always*
	professional social networking (LinkedIn)	*Never*	*Almost never*	*Sometimes*	*Almost always*	*Always*

DURATION OF USE

In a week, how much time of your university studies do you invest in…?						
**EDUCATIONAL**	blogs (Blogger, Wordpress…)	*Less than one hour*	*1-3 hours*	*3-6 hours*	*6-9 hours*	*More than 9 hours*
	wikis (Wikispaces, Mediawiki…)	*Less than one hour*	*1-3 hours*	*3-6 hours*	*6-9 hours*	*More than 9 hours*
	online word processing (Google Document…)	*Less than one hour*	*1-3 hours*	*3-6 hours*	*6-9 hours*	*More than 9 hours*
	online Presentations (Prezi, SlideShare, Google Presentations…)	*Less than one hour*	*1-3 hours*	*3-6 hours*	*6-9 hours*	*More than 9 hours*
	cloud storage (Drive, OneDrive, Dropbox…)	*Less than one hour*	*1-3 hours*	*3-6 hours*	*6-9 hours*	*More than 9 hours*
	online survey development (Forms Office, Google Forms, SurveyMonkey…)	*Less than one hour*	*1-3 hours*	*3-6 hours*	*6-9 hours*	*More than 9 hours*
	online response (Kahoot, Socrative, Poll Everywhere, Polldaddy…)	*Less than one hour*	*1-3 hours*	*3-6 hours*	*6-9 hours*	*More than 9 hours*
	online interactive notes (Pinterest, Lino It, Padlet…)	*Less than one hour*	*1-3 hours*	*3-6 hours*	*6-9 hours*	*More than 9 hours*
	recording (CamStudio, Screencast-O-Matic, Camtasia…)	*Less than one hour*	*1-3 hours*	*3-6 hours*	*6-9 hours*	*More than 9 hours*
	video conferencing (Skype, FaceTime, Hangouts…)	*Less than one hour*	*1-3 hours*	*3-6 hours*	*6-9 hours*	*More than 9 hours*
**SOCIAL**	synchronous communication (WhatsApp, Telegram…)	*Less than one hour*	*1-3 hours*	*3-6 hours*	*6-9 hours*	*More than 9 hours*
	social networking (Facebook…)	*Less than one hour*	*1-3 hours*	*3-6 hours*	*6-9 hours*	*More than 9 hours*
	image sharing (Instagram, Flickr, Picasa…)	*Less than one hour*	*1-3 hours*	*3-6 hours*	*6-9 hours*	*More than 9 hours*
	microblogging (Twitter)	*Less than one hour*	*1-3 hours*	*3-6 hours*	*6-9 hours*	*More than 9 hours*
**FUN**	online series and film viewing (Netflix, HBO, Amazon Prime…)	*Less than one hour*	*1-3 hours*	*3-6 hours*	*6-9 hours*	*More than 9 hours*
	video (YouTube, Vimeo…)	*Less than one hour*	*1-3 hours*	*3-6 hours*	*6-9 hours*	*More than 9 hours*
**PROFESSIONAL**	academic/research social networking (Academia, ResearchGate…)	*Less than one hour*	*1-3 hours*	*3-6 hours*	*6-9 hours*	*More than 9 hours*
	professional social networking (LinkedIn)	*Less than one hour*	*1-3 hours*	*3-6 hours*	*6-9 hours*	*More than 9 hours*

FEELING ABOUT USE

	How much fun do you find using… in your university studies?					
**EDUCATIONAL**	blogs (Blogger, Wordpress…)	*Not at all fun/ pleasurable*	*Not very fun/ pleasurable*	*Moderately fun/ pleasurable*	*Very fun/ pleasurable*	*Extremely fun/ pleasurable*
	wikis (Wikispaces, Mediawiki…)	*Not at all fun/ pleasurable*	*Not very fun/ pleasurable*	*Moderately fun/ pleasurable*	*Very fun/ pleasurable*	*Extremely fun/ pleasurable*
	online word processing (Google Document…)	*Not at all fun/ pleasurable*	*Not very fun/ pleasurable*	*Moderately fun/ pleasurable*	*Very fun/ pleasurable*	*Extremely fun/ pleasurable*
	online Presentations (Prezi, SlideShare, Google Presentations…)	*Not at all fun/ pleasurable*	*Not very fun/ pleasurable*	*Moderately fun/ pleasurable*	*Very fun/ pleasurable*	*Extremely fun/ pleasurable*
	cloud storage (Drive, OneDrive, Dropbox…)	*Not at all fun/ pleasurable*	*Not very fun/ pleasurable*	*Moderately fun/ pleasurable*	*Very fun/ pleasurable*	*Extremely fun/ pleasurable*
	online survey development (Forms Office, Google Forms, SurveyMonkey…)	*Not at all fun/ pleasurable*	*Not very fun/ pleasurable*	*Moderately fun/ pleasurable*	*Very fun/ pleasurable*	*Extremely fun/ pleasurable*
	online response (Kahoot, Socrative, Poll Everywhere, Polldaddy…)	*Not at all fun/ pleasurable*	*Not very fun/ pleasurable*	*Moderately fun/ pleasurable*	*Very fun/ pleasurable*	*Extremely fun/ pleasurable*
	online interactive notes (Pinterest, Lino It, Padlet…)	*Not at all fun/ pleasurable*	*Not very fun/ pleasurable*	*Moderately fun/ pleasurable*	*Very fun/ pleasurable*	*Extremely fun/ pleasurable*
	recording (CamStudio, Screencast-O-Matic, Camtasia…)	*Not at all fun/ pleasurable*	*Not very fun/ pleasurable*	*Moderately fun/ pleasurable*	*Very fun/ pleasurable*	*Extremely fun/ pleasurable*
	video conferencing (Skype, FaceTime, Hangouts…)	*Not at all fun/ pleasurable*	*Not very fun/ pleasurable*	*Moderately fun/ pleasurable*	*Very fun/ pleasurable*	*Extremely fun/ pleasurable*
**SOCIAL**	synchronous communication (WhatsApp, Telegram…)	*Not at all fun/ pleasurable*	*Not very fun/ pleasurable*	*Moderately fun/ pleasurable*	*Very fun/ pleasurable*	*Extremely fun/ pleasurable*
	social networking (Facebook…)	*Not at all fun/ pleasurable*	*Not very fun/ pleasurable*	*Moderately fun/ pleasurable*	*Very fun/ pleasurable*	*Extremely fun/ pleasurable*
	image sharing (Instagram, Flickr, Picasa…)	*Not at all fun/ pleasurable*	*Not very fun/ pleasurable*	*Moderately fun/ pleasurable*	*Very fun/ pleasurable*	*Extremely fun/ pleasurable*
	microblogging (Twitter)	*Not at all fun/ pleasurable*	*Not very fun/ pleasurable*	*Moderately fun/ pleasurable*	*Very fun/ pleasurable*	*Extremely fun/ pleasurable*
**FUN**	online series and film viewing (Netflix, HBO, Amazon Prime…)	*Not at all fun/ pleasurable*	*Not very fun/ pleasurable*	*Moderately fun/ pleasurable*	*Very fun/ pleasurable*	*Extremely fun/ pleasurable*
	video (YouTube, Vimeo…)	*Not at all fun/ pleasurable*	*Not very fun/ pleasurable*	*Moderately fun/ pleasurable*	*Very fun/ pleasurable*	*Extremely fun/ pleasurable*

**PROFESSIONAL**	academic/research social networking (Academia, ResearchGate...)	*Not at all fun/ pleasurable*	*Not very fun/ pleasurable*	*Moderately fun/ pleasurable*	*Very fun/ pleasurable*	*Extremely fun/ pleasurable*
	professional social networking (LinkedIn)	*Not at all fun/ pleasurable*	*Not very fun/ pleasurable*	*Moderately fun/ pleasurable*	*Very fun/ pleasurable*	*Extremely fun/ pleasurable*

ABILITY OR COMPETENCE

To what extent do you feel capable of (…) about / in your university studies?					
write a blog	Not at all competent	Not very competent	Moderately competent	Very competent	Extremely competent
write a wiki	Not at all competent	Not very competent	Moderately competent	Very competent	Extremely competent
make a video call	Not at all competent	Not very competent	Moderately competent	Very competent	Extremely competent
send a file / image through WhatsApp	Not at all competent	Not very competent	Moderately competent	Very competent	Extremely competent
watch videos about content related to your studies through YouTube	Not at all competent	Not very competent	Moderately competent	Very competent	Extremely competent
use a Prezi in a presentation	Not at all competent	Not very competent	Moderately competent	Very competent	Extremely competent
edit videos using Camtasia	Not at all competent	Not very competent	Moderately competent	Very competent	Extremely competent
vote questions through Socrative	Not at all competent	Not very competent	Moderately competent	Very competent	Extremely competent
design interactive notes	Not at all competent	Not very competent	Moderately competent	Very competent	Extremely competent
write tweets to your Twitter account	Not at all competent	Not very competent	Moderately competent	Very competent	Extremely competent
write comments on your Facebook wall	Not at all competent	Not very competent	Moderately competent	Very competent	Extremely competent
search for an article / scholarly journal on ResearchGate	Not at all competent	Not very competent	Moderately competent	Very competent	Extremely competent
search job openings through LinkedIn	Not at all competent	Not very competent	Moderately competent	Very competent	Extremely competent

IMPORTANTE OF BEING COMPETENT

To what extent do you consider it important to be able to carry out the following academic activities with a mobile device ...?					
access to library resources.	*Not at all important*	*Not very important*	*Moderately important*	*Very important*	*Extremely important*
view / check verify grades.	*Not at all important*	*Not very important*	*Moderately important*	*Very important*	*Extremely important*
sign up for courses.	*Not at all important*	*Not very important*	*Moderately important*	*Very important*	*Extremely important*
access information about events, student activities, clubs / organizations.	*Not at all important*	*Not very important*	*Moderately important*	*Very important*	*Extremely important*
Read texts.	*Not at all important*	*Not very important*	*Moderately important*	*Very important*	*Extremely important*
communicate with other students about academic topics outside of the classroom.	*Not at all important*	*Not very important*	*Moderately important*	*Very important*	*Extremely important*
seek information during classroom time.	*Not at all important*	*Not very important*	*Moderately important*	*Very important*	*Extremely important*
take notes.	*Not at all important*	*Not very important*	*Moderately important*	*Very important*	*Extremely important*
capture images of classroom activities and resources.	*Not at all important*	*Not very important*	*Moderately important*	*Very important*	*Extremely important*
record the teacher's class or classroom activities (audio, video or audio and video).	*Not at all important*	*Not very important*	*Moderately important*	*Very important*	*Extremely important*
cite sources.	*Not at all important*	*Not very important*	*Moderately important*	*Very important*	*Extremely important*
participate in interactive class activities.	*Not at all important*	*Not very important*	*Moderately important*	*Very important*	*Extremely important*
use the mobile phone to access university or identification services.	*Not at all important*	*Not very important*	*Moderately important*	*Very important*	*Extremely important*
to manage time.	*Not at all important*	*Not very important*	*Moderately important*	*Very important*	*Extremely important*
eliminate distractions.	*Not at all important*	*Not very important*	*Moderately important*	*Very important*	*Extremely important*
